# Football and Zumba Training in Female Hospital Staff: Effects after 12 and 40 Weeks on Self-Reported Health Status, Emotional Wellbeing, General Self-Efficacy and Sleep Problems

**DOI:** 10.3390/ijerph19031685

**Published:** 2022-02-01

**Authors:** Svein Barene, Peter Krustrup

**Affiliations:** 1 Department of Public Health and Sport Sciences, Inland Norway University of Applied Sciences, 2418 Elverum, Norway; 2 Department of Sports Science and Clinical Biomechanics, Sport and Health Sciences Cluster (SHSC), University of Southern Denmark, 5230 Odense, Denmark; pkrustrup@health.sdu.dk; 3 Danish Institute for Advanced Study (DIAS), University of Southern Denmark, 5230 Odense, Denmark; 4 Sport and Health Sciences, College of Life and Environmental Sciences, University of Exeter, Exeter EX1 2LU, UK

**Keywords:** female workers, self-perceived health, mood, sleep quality, soccer, dance

## Abstract

Background: This 40-weeks intervention study evaluates the effects on self-reported health status, emotional wellbeing, general self-efficacy and sleep problems among female hospital employees. Methods: 107 employees were cluster-randomized into three groups; football (FG), Zumba (ZG) and control (CG). Outcome measurements were conducted at baseline, 12 and 40 weeks. Weekly training frequencies in the first 12 and next 28 weeks were 2.4 ± 0.3 and 0.8 ± 0.2 in FG and 2.3 ± 0.3 and 0.9 ± 0.2 in ZG. Results: Compared to the CG, the ZG showed significant improvement in self-reported health status (*p* = 0.025), a reduced frequency of poor and restless sleep (on a 0–4 scale) (*p* = 0.004), as well as a tendency for reduction in the frequency of problems falling asleep (*p* = 0.055) and overall sleep problems (*p* = 0.051). Between baseline and 12 weeks, both the FG (*p* = 0.017) and the ZG (*p* = 0.017) showed within-group improvements in emotional wellbeing, whereas this improvement was maintained only in the ZG between baseline and 40 weeks (*p* = 0.002). The FG showed a significant within-group improvement in general self-efficacy (*p* = 0.012) between baseline and 12 weeks follow-up, with no such improvements in the other groups. Conclusion: The present findings revealed that a physical activity intervention with Zumba as well as football training may improve mental health and sleep outcomes in female hospital staff.

## 1. Introduction

A healthy nursing staff is crucial for optimal healthcare [1,2]. However, it has proved challenging to maintain good health among nurses, as this occupational group is considered as one of the most stressful occupations, comprising a relatively high proportion of high physical and mental work demands [3,4,5]. A worrying consequence of this is the increasing shortage of nurses worldwide [6], which emphasizes the importance of improving physical and mental health in this occupational group [7,8].

In general, a positive self-perceived health status is considered important for performing well at work [9], while poor self-assessed health is associated with the intention to leave the profession [10,11]. With reference to this, it is suggested that good emotional wellbeing may influence both positive adaptions to occupational stress, as well as satisfactory clinical competences in nurses [12], and that the ability of nurses to maintain their emotional wellbeing and to work effectively contributes to the retention of experienced staff [13]. Another factor regarding the importance for nurses’ mental health is self-efficacy, generally defined as judgment of ‘how well one can execute courses of action required to deal with prospective situations’ [14]. Previous studies have demonstrated that higher levels of self-efficacy, coupled with social support, facilitate healthier lifestyles and healthier coping behaviors for employees in high stress professions, such as nursing [15].

Poor sleep quality appears to be common in nursing staff, which partly has been explained by occupational stress [16,17] and work demands [18]. In previous, several studies have suggested that nurses’ sleep disorders not only influence their own health but also affect nursing quality and even the patients’ psychological health and treatment process [16,19,20]. Considering its negative impact on health, there is a need for effective measures that can help prevent sleep problems, in relation to sleep time and sleep perception, in this occupational group [21].

According to previous systematic reviews, it has been suggested that participation in football training may have positive effects on mental well-being among people with mental illness [22], whereas Zumba training is suggested to improve physical functioning in terms of quality of life [23]. Based on our knowledge, however, there are few previous intervention studies evaluating the effects of football and Zumba on psychological health outcomes among occupational groups with documented work-related mental health challenges. Through four previously published papers on football and Zumba training among female hospital staff, we have demonstrated positive effects of both training types on physical capacity and physiological health [24,25], musculoskeletal pain degree [26] as well as muscle strength, lean mass and postural balance [27]. Therefore, as previous research has indicated a positive association between physical activity and both self-reported health status [28,29], emotional wellbeing [30,31], self-efficacy [32] and sleep [33,34,35], the aim of the present study was to examine the effect from 12 and 40 weeks of football and Zumba training, respectively, on these outcomes among female hospital staff.

## 2. Materials and Methods

### 2.1. Study Design

The present 40-week randomized controlled training intervention among hospital employees at a larger Norwegian Hospital was performed between January and October 2011. The inclusion criteria for participation were hospital employees of either sex aged 25 to 65 years, with pregnancy, angina pectoris and life-threatening diseases as exclusion criteria. However, due to a minority of participating males, only females were included in the statistical analyses. The project, and all participants, gave their written informed consent to participate in the study.

### 2.2. Recruitment of Participants

**Figure 1:**
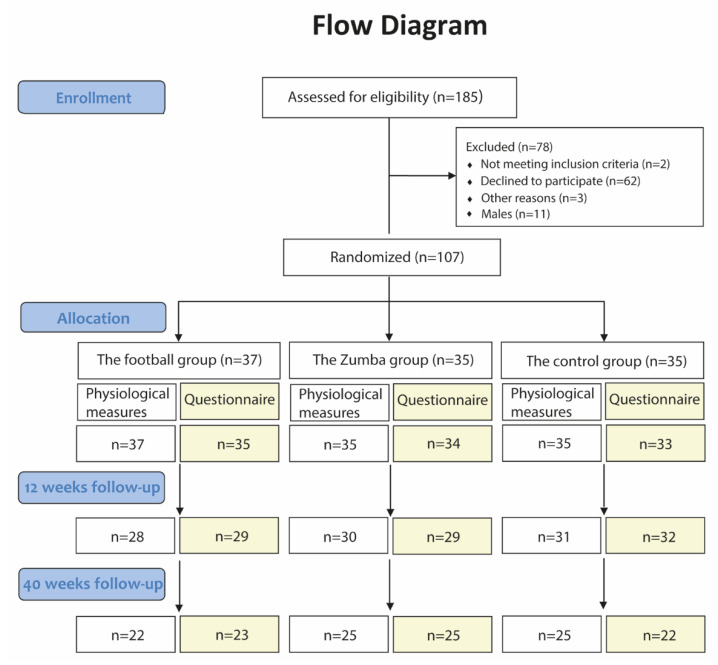
Flow diagram showing the number of participants initially enrolled in the study and randomized to the three groups, as well as the number of drop-outs throughout the 40 weeks intervention period related to (i) the objective physiological measurements and (ii) the online questionnaire, respectively.

The recruitment process was previously presented [24]. In short, out of a total of 660 female hospital staff, 161 completed the initial screening questionnaire. Of these, 109 females completed the baseline tests, whereas 107 fulfilled the inclusion criteria and consented to participate in the study with subsequent random allocation either to a football group, a Zumba group or a control group (Figure 1).

### 2.3. Randomization Procedure

The randomization procedure was previously reported [24]. In short, the department with the largest number of participants (n = 28) formed cluster 1, which became the basis for the composition of cluster 2 (n = 27) and cluster 3 (n = 28) that were matched on sex, BMI, age and work seniority. The remaining consenters were assigned into three smaller clusters, cluster A (n = 7), B (n = 8) and C (n = 9) and were matched on the same variables as above. The randomization was performed by the project manager by drawing lots according to the following procedure; the football group: cluster 3 + C (n = 37), the Zumba group: cluster 2 + B (n = 35) and the control group: cluster 1 + A (n = 35) [24].

### 2.4. Intervention Content

The design of the training intervention has been described in previous work [25]. Briefly, all training sessions were conducted outside working hours. During the first 12 weeks, both intervention groups were offered three 1 h training sessions per week, with the opportunity of two 1 h sessions during the last 28 weeks. Participants in both intervention groups had little or no previous experience with the activities. The football sessions were facilitated as small-sided games in a traditional gymnastics hall (10 m × 20 m) at the hospital, as well as in a municipal sports hall (20 m × 40 m) located close to the hospital. The Zumba sessions were conducted at a fitness center close to the hospital and were supervised by certified instructors.

### 2.5. Measurement Procedures Pre, during and Post the Intervention

The participants were invited to take several measurements at baseline (January 2011) and at follow-up tests after 12 (April 2011) and 40 weeks (October 2011), respectively. In addition to the online questionnaire, all of the three test rounds consisted of the following five measurements (followed by reference to the respective papers in parentheses):

(1) Venous blood samples obtained from the antecubital vein using Vacuette EDTA and serum tubes (Greiner Bio-One, Kremsmünster, Austria). Serum glucose, triglycerides, total cholesterol, direct LDL-cholesterol and HDL-cholesterol were analyzed on an ADVIA 1800 instrument from Siemens (Siemens Diagnostics, Tarrytown, NY, USA). Serum osteocalcin was quantified using a commercially available enzyme-linked immunosorbent assay kit with antibodies specific to the N-terminal region and N-terminal-mid fragment amino acid sequences (Immunodiagnostic Systems Ltd., Boldon, UK). Serum leptin was measured using AlphaLISA assay kits (Perkin Elmer, Waltham, MA, USA), with luminescence recorded using an EnSpire 2300-001L plate reader (Perkin Elmer) [24,25].

(2) Total body and regional fat, muscle, and bone mass were determined by dual-energy X-ray absorptiometry scans (QDR Discovery Wi, Hologic Inc, Bedford, MA, USA) [24,25].

(3) Systolic and diastolic blood pressure was measured after 10 min resting in a supine position on the left arm by an automatic upper arm blood pressure monitor (HEM-709; OMRON, Vernon Hills, IL, USA). An average from the two (of a total of four) lowest values of blood pressure was used for the statistical analyses [24,25].

(4) Pulmonary gas exchange (VMAX Spectra Series, SensorMedics Corporation, Yorba Linda, CA, USA), heart rate (Polar Team System, Polar Electro Oy, Kempele, Finland) and blood lactate (LactatePro™ LT-1710, ARKRAY, Inc, Kyoto, Japan) were obtained during a standardized bicycle test with submaximal cycling for 6 min (100 W), followed by a 2 min rest period, and then a test to exhaustion starting with 2 min at 80 W followed by increments of 30 W/30 s. The VO_2_ peak and maximal heart rate was determined as the peak value reached in a 40 s and 15 s period, respectively, during the last part of the incremental test [24,25].

(5) Isometric muscle strength was measured with Newtest Isometric Force System dynamometer (Newtest, Oy, Oulu, Finland), and included measurements of neck extension, trunk flexion and extension and leg extension. Measurements of the maximal jump height were performed on a force platform (OR6-5-2000, AMTI, Watertown, MA, USA), and data were collected at 1000 Hz, with the real time displayed and saved with the use of computer software (NetForce 2.0, AMTI, Watertown, MA, USA) for later analyses [27].

### 2.6. Self-Reported Health Status

Self-rated health was measured using the single-item question: ‘How would you rate your health in general?’ derived from the Rand 36-Item Health Survey [36] containing a 5-point Likert scale with the following options: ‘poor’ (1), ‘fair’ (2), ‘good’ (3), ‘very good’ (4), and ‘excellent’ (5).

### 2.7. Emotional Wellbeing

The measure of emotional wellbeing was derived from the RAND 36-item Health Survey [36], comprising the following five items: (i) ‘have you been a very nervous person?’, (ii) ‘have you felt so down in the dumps that nothing could cheer you up?’, (iii) ‘have you felt calm and peaceful?’, (iv) ‘have you felt downhearted and blue?’, and (v) ‘have you been a happy person?’. Each item contained a 5-point Likert scale with the subsequent options: (i) ‘all of the time’ (1), (ii) ‘most of the time’ (2), (iii) ‘a good bit of the time’ (3), (iv) ‘some of the time’ (4), (v) ‘a little bit of the time’ (5), and (vi) ‘none of the time’ (6). Prior to averaging the items, they were recoded as follows; items (ii), (iii) and (iv): 1 = 0%, 2 = 20%, 3 = 40%, 4 = 60%, 5 = 80% and 6 = 100%, whereas items (i) and (iv) were oppositely recoded, i.e.,: 1 = 100%, 2 = 80%, 3 = 60%, 4 = 40%, 5 = 20%, and 6 = 100%. A high average score defines a more favorable emotional wellbeing on a scale from 0 to 100%.

### 2.8. General Self-Efficacy

To be able to examine the effects on general self-efficacy, the three following items from the original ten-item scale by Schwarzer [37] were used: (i) ‘I feel confident that I can handle unexpected events’, (ii) ‘when I have a problem, I can usually find several ways of solving it’, and (iii) ‘regardless of what happens, I usually manage’. Each item contained a 5-point Likert scale with the following options: ‘never’ (1), ‘rarely’ (2), ‘sometimes’ (3), ‘often’ (4), and ‘always’ (5). A total score was calculated based on the average score of the three items.

### 2.9. Sleep

Self-reported sleep problems during the past three months were assessed using the following four single-items derived from a modified version of the Karolinska Sleep Questionnaire [38]: (i) ‘how often did you have problems falling asleep?’, (ii) ‘how often did you wake up too early and could not fall asleep again?’, (iii) ‘how often did you wake up several times and were unable to fall asleep again?’, and (iv) ‘how often was your sleep poor and disturbed?’ [39]. The disturbed sleep score was ranged on 5-point Likert scale comprising the following options: ‘never’ (1), ‘rarely’ (2), ‘sometimes’ (3), ‘very often’ (4), and ‘always’ (5). High scores represent poorer sleep. In addition to the four single-item scales, the total score of sleep problems was calculated as the average score of the four items.

### 2.10. Statistical Analyses

All statistical analyses were performed using SPSS version 27.0. Prior to the study, an effect calculation was performed for the primary outcome, VO_2_ peak. The power was set to 0.8 with an alpha level of 0.05. Based on previous similar studies [28,40,41], the effect size was set at 5% with a variation of the effect of 10. The estimates revealed that 32 participants were needed in each group for comparison between each respective intervention group and the control group. Our main objective was to evaluate the between-group differences on the dependent variables comparing the intervention groups, i.e., the football group and the Zumba groups, respectively, with the control group. The linear mixed model analyses were used to model each outcome measure at three time points [42], i.e., baseline, 12 weeks and 40 weeks. In the analyses, the delta values calculated between the three timepoints were used as the dependent variables. Due to a tendency for difference in the baseline body mass index (BMI) between one of the intervention groups and the control group, BMI was used as a fixed factor, in addition to the baseline values for the best model-fit [43]. All results are reported as descriptive data and 95% CIs for all possible comparisons, with *p* < 0.05 defined as the level of statistical significance.

## 3. Results

### 3.1. Baseline Characteristics

The age, body mass index (BMI) and job seniority of the participants included in the baseline questionnaire were, on average, 46.1 ± 9.2 years, 25.4 ± 3.1 kg/m^2^ and 74.5 ± 64.7 months, respectively (Table 1). No significant between-group differences were observed at baseline. Self-reported physical activity level the past 12 months measured on a Likert scale from 1 (being almost completely inactive) to 4 (regular hard physical training several times per week) revealed an overall average of 2.5 ± 0.6, with no significant between-group differences (*p* = 0.68)

### 3.2. Training Frequency

As reported in a previous paper [24], the total number of training sessions during the first 12 weeks were 28.2 ± 6.0 (2.4 ± 0.5 per week) vs. 27.7 ± 4.0 (2.3 ± 0.3 per week) in the football group and the Zumba group, respectively, whereas the corresponding participation during the last 28 weeks in the two respective groups were 24.4 ± 5.7 (0.9 ± 0.2 per week) vs. 19.8 ± 4.9 (0.8 ± 0.2 per week) [25]. This corresponds to a reduced training frequency of 63% and 65% in the football group and the Zumba group, respectively.

### 3.3. Self-Reported Health Status

**Figure 2:**
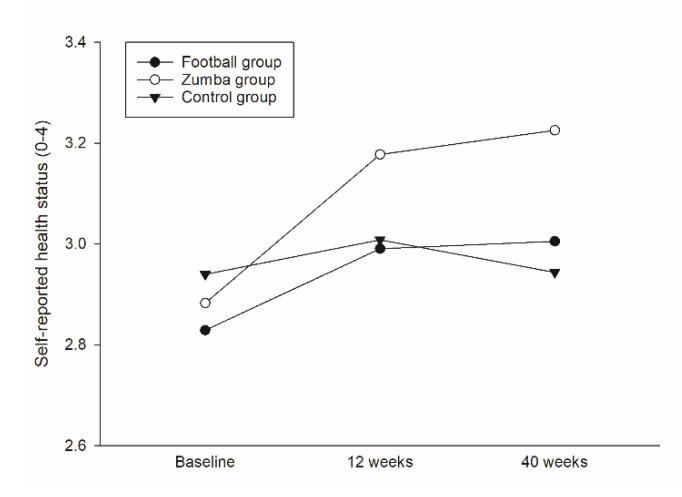
Between-group differences in self-reported health status over 12 and 40 weeks.

**Figure 3:**
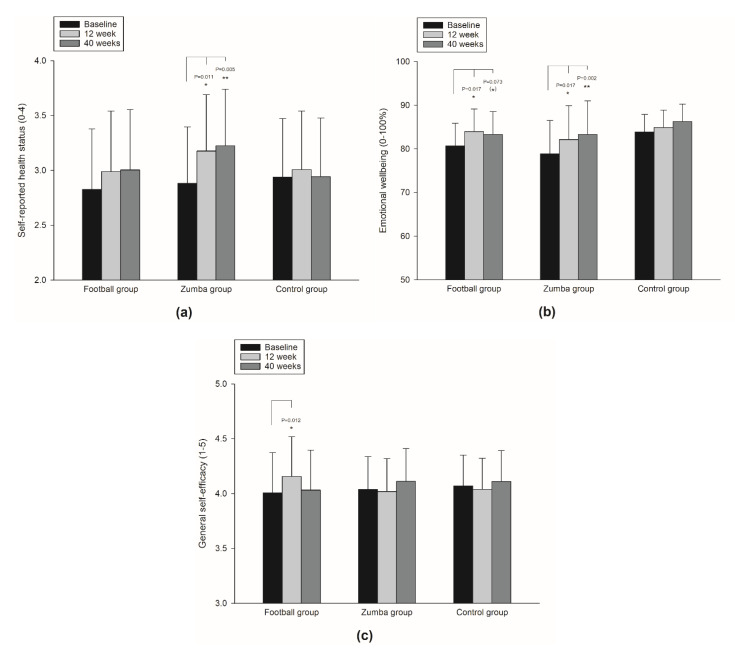
Within-group changes over 12 and 40 weeks in self-reported health status (**a**); emotional wellbeing (**b**); and general self-efficacy (**c**). Significant within-group change denotes; ** (*p* < 0.01), * (*p* < 0.05), ^(^*^)^ (*p* < 0.1) tendency for change.

Based on the linear mixed model analyses, the Zumba group revealed a significant higher overall mean in self-reported health status (on a Likert scale from 0–4) during the 40 weeks intervention period in comparison to the controls (0.16, 95% CI 0.02 to 0.30, *p* = 0.025), with no such improvement in the football group compared to the controls (*p* = 0.585) (Table 2/Figure 2). In the Zumba group, a corresponding within-group increase in self-reported health status was observed both between baseline and 12 (0.30, 95% CI 0.07 to 0.52, *p* = 0.011) and 40 (0.34, 95% CI 0.11 to 0.58, *p* = 0.005) weeks (Figure 3a), respectively, whereas no such within-group changes were found for either the football group or the controls (Table 2).

### 3.4. Emotional Wellbeing

With regard to the multi-item scaled measure of emotional wellbeing, no significant between-group difference in the overall means was observed for either of the intervention groups compared to the controls, i.e., −0.10, 95% CI −1.81 to 1.62 (*p* = 0.913) and −0.24, 95% CI −1.94 to 1.47 (*p* = 0.786) in the football and the Zumba groups, respectively (Table 2). However, between baseline and 12 weeks, both the football group (3.27, 95% CI 0.58 to 5.95, *p* = 0.017) and the Zumba group (3.30, 95% CI 0.60 to 6.00, *p* = 0.017) revealed significant within-group changes (Figure 3b), with no such improvement in the controls (Table 2). Between baseline and 40 weeks, only the Zumba group showed a significant within-group change (4.44, 95% CI 1.62 to 7.26, *p* = 0.002), with a tendency for improvement in the football group (2.63, 95% CI −0.25 to 5.51, *p* = 0.073) (Figure 3b).

### 3.5. General Self-Efficacy

In comparison to the controls, no between-group differences were observed in terms of general self-efficacy (on a Likert scale from 1–5) for either the football group (0.03, 95% CI −0.04 to 0.10, *p* = 0.375) or the Zumba group (0.00, 95% CI −0.07 to 0.07, *p* = 0.933) (Table 2). However, in the football group a significant within-group change was observed between baseline and 12 weeks (0.15, 95% CI 0.03 to 0.26, *p* = 0.012) (Figure 3c), with no such within-group change observed in any of the other groups (Table 2).

### 3.6. Sleep

**Figure 4:**
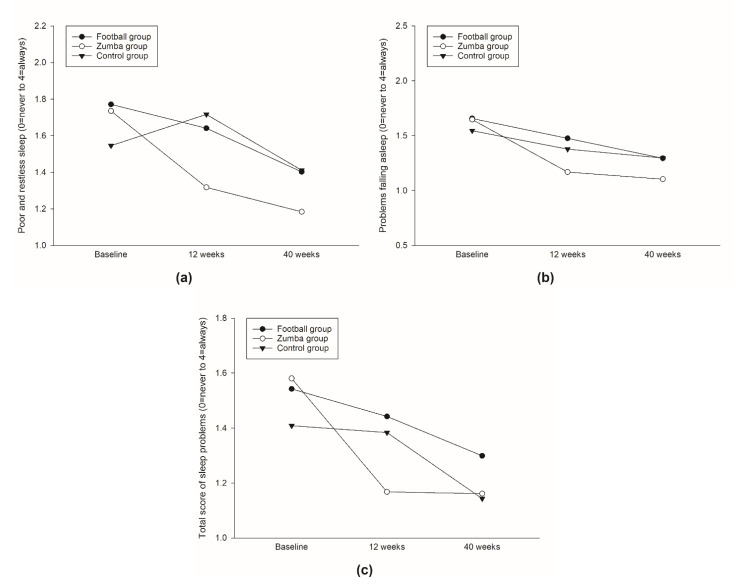
Between-group differences over 12 and 40 weeks in self-reported frequency of poor and restless sleep (**a**); frequency of problems falling asleep (**b**); and total score of sleep problems (**c**).

With regards to the frequency of poor and restless sleep during the past three months, the Zumba group significantly reduced the overall mean (on a 0–4 scale) over the 40 week intervention period compared to the controls (−0.27, 95% CI −0.45 to −0.09, *p* = 0.004) (Figure 4a), with no such difference between the football group and the control group (*p* = 0.296) (Table 2). In the Zumba group, this was supported by significant within-group decreases both between baseline and 12 weeks (*p* < 0.01), as well as between baseline and 40 weeks (*p* < 0.001). In addition, the football group revealed a significant within-group decrease in poor and restless sleep between baseline and 40 weeks (*p* < 0.05) (Table 2).

Furthermore, similar tendencies for between-group differences in the overall mean (on 0–4 scales) in favor of the Zumba group compared with the controls were revealed in the frequency of problems falling asleep (−0.17, 95% CI −0.33 to 0.00, *p* = 0.055) (Figure 4b), as well as in the total score of sleep problems (−0.14, 95% CI −0.29 to 0.00, *p* = 0.051) in the past three months (Figure 4c) over the 40-week intervention period. In the Zumba group these tendencies were supported by significant within-group reductions in both the frequencies of problems falling asleep (*p* < 0.001) and total sleep problems (*p* < 0.001) between baseline and 12 weeks and 40 weeks (Table 2). No between-group differences in either poor and restless sleep or in the total score of sleep problems in the past three months were observed in comparison to the football group and the controls (Table 2). However, the football group revealed significant within-group reduction in the frequency of problems falling asleep between baseline and 40 weeks (*p* < 0.05), without any improvements related to total score of sleep problems (Table 2).

**Figure 5:**
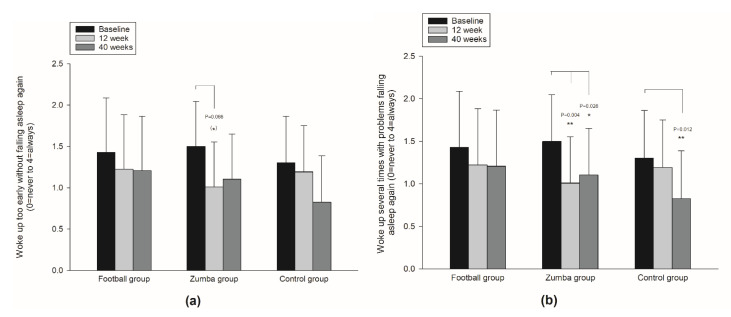
Within-group changes over 12 and 40 weeks in frequency of waking up too early without falling asleep again (**a**); and frequency of waking up several times with problems falling asleep again (**b**). Significant within-group change denotes; ** (*p* < 0.01), * (*p* < 0.05), ^(^*^)^ (*p* < 0.1) tendency for change.

No between-group differences in overall mean (on a 0–4 scale) were observed in either the frequency of waking up early without falling asleep again or the frequency of waking up several times during night with problems falling asleep again (Table 2). However, in the Zumba group, a tendency for reduction in the frequency of waking up too early without falling asleep again was observed between baseline and 12 weeks (*p* < 0.1) (Figure 5a), including significant within-group reductions in the frequency of waking up several times with problems falling asleep again both between baseline and 12 weeks (*p* < 0.01), as well as between baseline and 40 weeks (*p* < 0.05) (Figure 5b/Table 2).

## 4. Discussion

The main findings of the present study were that the participants in the Zumba group improved their overall mean score on self-reported health status, as well as reduced the frequency of poor and restless sleep, during the 40 weeks intervention period in comparison to the controls. With regards to emotional wellbeing, both the football group and the Zumba group showed significant improvements from baseline to 12 weeks follow-up, whereas only the Zumba group had a corresponding improvement between baseline and 40 weeks. Between baseline and 12 weeks, a significant within-group improvement in general self-efficacy was observed in the football group only. It should be emphasized that the training frequency was reduced by 65% and 63% for the Zumba and football group in the last 28 weeks compared to the first 12 weeks and that this may have affected the study outcomes.

In favor of the Zumba group, the linear mixed model analyses revealed a significant between-group difference in self-reported health status compared to the controls, which was supported by corresponding significant within-group changes between baseline and the two respective follow-ups in the Zumba group. This is in accordance with a previous nine months worksite physical exercise intervention comprising 1 h bi-weekly sessions (aerobic fitness and strength training), and also demonstrating improvement on perceived health status in female home care workers [28], as well as to previous cross-sectional studies demonstrating significant associations between recreational physical activity and self-reported health status [29,44,45].

With regards to general self-efficacy, no between-group differences were found for either of the intervention groups compared to the controls. However, the football group showed a significant within-group improvement between baseline and 12 weeks, which is in accordance with previous prospective studies, suggesting that physical activity may improve self-efficacy that, in turn, can lead to an increased perceived quality of life [32,46,47]. According to Bandura (1977), an improvement in self-efficacy will lead to previous barriers being considered as challenges [48]. However, the improvement in general efficacy in the football group was not maintained at 40 weeks of follow-up, which may be due to a ~63% reduction in training frequency when comparing the first 12 weeks with the last 28 weeks of the intervention.

In terms of sleep, the baseline measurements on self-reported sleep quality showed that our sample of female hospital staff did not suffer from sleep problems that have often been reported for this occupational group in previous studies [49,50]. Nonetheless, the Zumba group showed a significant reduction in the frequency of poor and restless sleep (on a 0–4 scale), as well as a clear tendency for a reduction in total sleep problems compared to the controls (*p* = 0.051), with corresponding within-group reductions between baseline and 12 and 40 weeks, respectively (*p* < 0.001). This is in line with a previous meta-analytical review reporting the moderate-to-strong positive effects of regular physical activity on overall sleep quality, and small-to-moderate beneficial effect on sleep onset latency [51]. Apart from within-group improvements (*p* < 0.05) in two of the four single-item questions, as well as a tendency for within-group improvement in total sleep between baseline and 40 weeks follow-up, the overall improvement in sleep was not as prominent for the football group compared to the Zumba group. In a previous cross-sectional study examining associations between self-reported sleep quality and exercise intensity, a significant lower prevalence of sleep problems was observed in those with moderate intensity levels, while there was no effect on sleep at either low or high intensity [34]. Given that the average intensity level was significantly higher (*p* < 0.05) in the football group compared to the Zumba group in the present study, this could potentially be an explanation for the lack of improvement in sleep in the football group.

### Strength and Limitations

One of the strengths of the study is the use of randomized controlled design together with a relatively high response rate to the survey at the three respective measurement points at baseline (95%), 12 (84%) and 40 (64%) week follow-up. Potential limitations are the use of self-reported data and lack of control over the participants’ physical activity level during leisure which may represent bias to the results. In addition, the inclusion of females only in the statistical analyses could potentially constitute a weakness of the study. However, given that health care workers are a predominantly female workforce, as well as the low number of male participants (n = 11) with low probability of influencing the outcome variables, we consider this to be of minor importance. An additional potential weakness of the study is the lack of information on dietary habits, which is suggested to be associated with, for example, sleep quality.

## 5. Conclusions

The present study indicates that 40 weeks of Zumba training may improve perceived health status and sleep. Moreover, significant within-group improvements in general self-efficacy were observed for the football group after 12 weeks, whereas both the football group and the Zumba group showed significant within-group improvements in emotional wellbeing throughout the intervention period. Future studies may reveal whether larger long-term effects would be seen if the training frequency was kept high throughout the training intervention period.

## Figures and Tables

**Table 1:** Subject characteristics at baseline. The table presents age, anthropometry, physical activity level, self-reported health status, perceived emotional wellbeing, general self-efficacy, as well as self-reported sleep problems for the football group (*n* = 35), the Zumba group (*n* = 34), and the control group (*n* = 34), respectively. The *p*-values refer to comparisons between all groups.

Characteristics	Football Group		Zumba Group		Control Group		Total		*p*-Value
	Mean	SD	Mean	SD	Mean	SD	Mean	SD	
Age (years)	44.8	8.9	46.3	9.5	47.2	9.4	46.1	9.2	0.56
Body mass index (kg/m^2^)	24.9	3.1	25.3	2.7	26.0	3.6	25.4	3.1	0.36
Physical activity level (1–4 scale)	2.5	0.6	2.5	0.7	2.6	0.6	2.5	0.6	0.68
Self-reported health status (0–4 scale)	2.83	0.79	2.88	0.73	2.97	0.76	2.89	0.75	0.74
Emotional wellbeing (0–100%)	80.7	8.5	78.9	12.4	83.8	6.4	81.1	9.5	0.10
General self-efficacy (1–5 scale)	4.01	0.46	4.04	0.37	4.09	0.36	4.05	0.40	0.71
Sleep problems past 3 months (0–4 scale)									
Poor and restless sleep	1.31	0.96	1.44	1.16	1.24	0.92	1.33	1.01	0.70
Problems falling asleep	1.66	1.00	1.65	0.88	1.53	1.11	1.61	0.99	0.84
Woke up too early without falling asleep again	1.43	1.04	1.50	0.86	1.29	0.87	1.41	0.92	0.65
Woke up several times and unable to fall asleep again	1.77	0.88	1.74	0.83	1.53	0.96	1.68	0.89	0.48
Total score of sleep problems	1.54	0.78	1.58	0.75	1.40	0.83	1.51	0.78	0.60

**Table 2:** Within-group changes and between-group differences in the soccer group (SG = 35), the Zumba group (ZG = 34) and the control group (CG = 34) during 12 and 40 weeks of training.

Characteristics	Within-Group Changes						Between-Group Differences					
	SG		ZG		CG		SG Group vs. CG			ZG vs. CG		
	Change	95% CI	Change	95% CI	Change	95% CI	Diff.	95% CI	Sig.	Diff.	95% CI	*p*-Value
Self-reported health (0–4 scale)												
0–12 weeks	0.16	−0.07 to 0.39	0.30 *	0.07 to 0.52	0.07	−0.16 to 0.30	0.04	−0.10 to 0.18	0.585	0.16	0.02 to 0.30	0.025
0–40 weeks	0.18	−0.07 to 0.42	0.34 **	0.11 to 0.58	0.00	−0.25 to 0.26						
Emotional wellbeing (0–100%)												
0–12 weeks	3.27 *	0.58 to 5.95	3.30 *	0.60 to 6.00	0.99	−1.69 to 3.67	−0.10	−1.81 to 1.62	0.913	−0.24	−1.94 to 1.47	0.786
0–40 weeks	2.63 ^(^*^)^	−0.25 to 5.51	4.44 **	1.62 to 7.26	2.36	−0.62 to 5.35						
General self-efficacy (1–5 scale)												
0–12 weeks	0.15 *	0.03 to 0.26	−0.02	−0.13 to 0.10	−0.03	−0.14 to 0.08	0.03	−0.04 to 0.10	0.375	0.00	−0.07 to 0.07	0.933
0–40 weeks	0.02	−0.10 to 0.15	0.08	−0.05 to 0.20	0.04	−0.09 to 0.17						
Sleep problems past 3 months (0–4 scale)												
Poor and restless sleep												
0–12 weeks	−0.13	−0.43 to 0.16	−0.42 **	−0.71 to −0.12	0.17	−0.12 to 0.47	−0.10	−0.29 to 0.09	0.296	−0.27	−0.45 to −0.09	0.004
0–40 weeks	−0.37 *	−0.69 to −0.05	−0.55 ***	−0.86 to −0.24	−0.14	−0.46 to 0.19						
Problems falling asleep												
0–12 weeks	−0.18	−0.45 to 0.09	−0.48 ***	−0.75 to −0.20	−0.17	−0.44 to 0.10	0.00	−0.17 to 0.18	0.973	−0.17	−0.33 to 0.00	0.055
0–40 weeks	−0.36 *	−0.66 to −0.07	−0.54 ***	−0.83 to −0.26	−0.25	−0.55 to 0.06						
Woke up too early without falling asleep again												
0–12 weeks	0.08	−0.24 to 0.39	−0.30 ^(^*^)^	−0.62 to 0.02	0.01	−0.30 to 0.33	0.06	−0.14 to 0.26	0.525	−0.08	−0.28 to 0.12	0.423
0–40 weeks	−0.07	−0.41 to 0.27	−0.22	−0.55 to 0.11	−0.14	−0.50 to 0.21						
Woke up several times with problems falling asleep again												
0–12 weeks	−0.20	−0.54 to 0.13	−0.49 **	−0.83 to −0.16	−0.11	−0.44 to 0.22	0.10	−0.11 to 0.31	0.357	−0.03	−0.24 to 0.18	0.771
0–40 weeks	−0.22	−0.58 to 0.14	−0.40 *	−0.74 to −0.05	−0.48 *	−0.85 to −0.11						
Total score of sleep problems												
0–12 weeks	−0.10	−0.33 to 0.13	−0.41 ***	−0.65 to −0.18	−0.03	−0.26 to 0.21	0.01	−0.13 to 0.16	0.865	−0.14	−0.29 to 0.00	0.051
0–40 weeks	−0.24 ^(^*^)^	−0.49 to 0.00	−0.42 ***	−0.66 to −0.18	−0.27 *	−0.52 to −0.01						

Within-group data are presented as mean change (95% CI) and between-group data as estimated overall mean difference (95% CI). Significant within-group change denotes; *** (*p* < 0.001), ** (*p* < 0.01), * (*p* < 0.05), ^(^*^)^ (*p* < 0.1) tendency for change.

## Data Availability

The datasets generated during and/or analyzed during the current study are available from the corresponding author on reasonable request.

## References

[1] Penque S. (2019). Mindfulness to promote nurses’ well-being. Nurs. Manag..

[2] Brand S.L., Thompson Coon J., Fleming L.E., Carroll L., Bethel A., Wyatt K. (2017). Whole-system approaches to improving the health and wellbeing of healthcare workers: A systematic review. PLoS ONE.

[3] Jamali J., Roustaei N., Ayatollahi S.M.T., Sadeghi E. (2015). Factors Affecting Minor Psychiatric Disorder in Southern Iranian Nurses: A Latent Class Regression Analysis. Nurs. Midwifery Stud..

[4] Pisaniello S.L., Winefield H.R., Delfabbro P.H. (2012). The influence of emotional labour and emotional work on the occupational health and wellbeing of South Australian hospital nurses. J. Vocat. Behav..

[5] Mohanty A., Kabi A., Mohanty A.P. (2019). Health problems in healthcare workers: A review. J Fam. Med. Prim. Care.

[6] Lin P.-Y., MacLennan S., Hunt N., Cox T. (2015). The influences of nursing transformational leadership style on the quality of nurses’ working lives in Taiwan: A cross-sectional quantitative study. BMC Nurs..

[7] Gao T., Ding X., Chai J., Zhang Z., Zhang H., Kong Y., Mei S. (2017). The influence of resilience on mental health: The role of general well-being. Int. J. Nurs. Pract..

[8] Yu F., Cavadino A., Mackay L., Ward K., King A., Smith M. (2020). Physical activity and personal factors associated with nurse resilience in intensive care units. J. Clin. Nurs..

[9] De Oliveira D.R., Griep R.H., Portela L.F., Rotenberg L. (2017). Intention to leave profession, psychosocial environment and self-rated health among registered nurses from large hospitals in Brazil: A cross-sectional study. BMC Health Serv. Res..

[10] Hasselhorn H.-M., Tackenberg P., Kuemmerling A., Wittenberg J., Simon M., Conway P., Bertazzi P., Beermann B., Büscher A., Camerino D. (2006). Nurses’ health, age and the wish to leave the profession--findings from the European NEXT-Study. La Med. Del Lav..

[11] Josephson M., Lindberg P., Voss M., Alfredsson L., Vingård E. (2008). The same factors influence job turnover and long spells of sick leave—a 3-year follow-up of Swedish nurses. Eur. J. Public Health.

[12] Cruz J.P. (2017). Quality of life and its influence on clinical competence among nurses: A self-reported study. J. Clin. Nurs..

[13] Siffleet J., Williams A.M., Rapley P., Slatyer S. (2015). Delivering best care and maintaining emotional wellbeing in the intensive care unit: The perspective of experienced nurses. Appl. Nurs. Res..

[14] Bandura A. (1982). Self-efficacy mechanism in human agency. Am. Psychol..

[15] Jordan T.R., Khubchandani J., Wiblishauser M. (2016). The Impact of Perceived Stress and Coping Adequacy on the Health of Nurses: A Pilot Investigation. Nurs. Res. Pract..

[16] Han Y., Yuan Y., Zhang L., Fu Y. (2016). Sleep disorder status of nurses in general hospitals and its influencing factors. Psychiatr. Danub..

[17] Rocha M.C.P.D., Martino M.M.F.D. (2010). Stress and sleep quality of nurses working different hospital shifts. Rev. Esc. Enferm. USP.

[18] Geiger-Brown J., Trinkoff A., Rogers V.E. (2011). The Impact of Work Schedules, Home, and Work Demands on Self-Reported Sleep in Registered Nurses. J. Occup. Environ. Med..

[19] Feleke S.A., Mulatu M.A., Yesmaw Y.S. (2015). Medication administration error: Magnitude and associated factors among nurses in Ethiopia. BMC Nurs..

[20] Tarhan M., Aydın A., Ersoy E., Dalar L. (2018). The sleep quality of nurses and its influencing factors. Eurasian J. Pulmonol..

[21] Zeng L.-N., Yang Y., Wang C., Li X.-H., Xiang Y.-F., Hall B.J., Ungvari G.S., Li C.-Y., Chen C., Chen L.-G. (2020). Prevalence of Poor Sleep Quality in Nursing Staff: A Meta-Analysis of Observational Studies. Behav. Sleep Med..

[22] Friedrich B., Mason O.J. (2017). “What is the score?” A review of football-based public mental health interventions. J. Public Ment. Health.

[23] Vendramin B., Bergamin M., Gobbo S., Cugusi L., Duregon F., Bullo V., Zaccaria M., Neunhaeuserer D., Ermolao A. (2016). Health Benefits of Zumba Fitness Training: A Systematic Review. PM&R.

[24] Barene S., Krustrup P., Jackman S.R., Brekke O.L., Holtermann A. (2013). Do soccer and Zumba exercise improve fitness and indicators of health among female hospital employees? A 12-week RCT. Scand. J. Med. Sci. Sports.

[25] Barene S., Krustrup P., Brekke O.L., Holtermann A. (2014). Soccer and Zumba as health-promoting activities among female hospital employees: A 40-weeks cluster randomised intervention study. J. Sports Sci..

[26] Barene S., Krustrup P., Holtermann A. (2014). Effects of the workplace health promotion activities soccer and Zumba on muscle pain, work ability and perceived physical exertion among female hospital employees. PLoS ONE.

[27] Barene S., Holtermann A., Oseland H., Brekke O.-L., Krustrup P. (2016). Effects on muscle strength, maximal jump height, flexibility and postural sway after soccer and Zumba exercise among female hospital employees: A 9-month randomised controlled trial. J. Sports Sci..

[28] Pohjonen T., Ranta R. (2001). Effects of Worksite Physical Exercise Intervention on Physical Fitness, Perceived Health Status, and Work Ability among Home Care Workers: Five-Year Follow-up. Prev. Med..

[29] Kaleta D., Makowiec-Dabrowska T., Dziankowska-Zaborszczyk E., Jegier A. (2006). Physical activity and self-perceived health status. Int. J. Occup. Med. Environ. Health.

[30] Penedo F.J., Dahn J.R. (2005). Exercise and well-being: A review of mental and physical health benefits associated with physical activity. Curr. Opin. Psychiatry.

[31] Aparicio V.A., Flor-Alemany M., Marín-Jiménez N., Coll-Risco I., Aranda P. (2021). A 16-week concurrent exercise program improves emotional well-being and emotional distress in middle-aged women: The FLAMENCO project randomized controlled trial. Menopause.

[32] Elavsky S., McAuley E., Motl R.W., Konopack J.F., Marquez D.X., Hu L., Jerome G.J., Diener E. (2005). Physical activity enhances long-term quality of life in older adults: Efficacy, esteem, and affective influences. Ann. Behav. Med..

[33] Lang C., Brand S., Feldmeth A.K., Holsboer-Trachsler E., Pühse U., Gerber M. (2013). Increased self-reported and objectively assessed physical activity predict sleep quality among adolescents. Physiol. Behav..

[34] Litleskare S., Vaktskjold A., Barene S. (2018). A cross-sectional study to examine the association between self-reported sleep and the frequency, duration and intensity of exercise. J. Sports Med. Phys. Fit..

[35] Yang P.-Y., Ho K.-H., Chen H.-C., Chien M.-Y. (2012). Exercise training improves sleep quality in middle-aged and older adults with sleep problems: A systematic review. J. Physiother..

[36] Hays R.D., Sherbourne C.D., Mazel R.M. (1993). The rand 36-item health survey 1.0. Health Econ..

[37] Schwarzer R. (1993). Measurement of perceived self-efficacy: Psychometric scales for cross-cultural research.

[38] Akerstedt T., Hume K., Minors D., Waterhouse J. (1994). The meaning of good sleep: A longitudinal study of polysomnography and subjective sleep quality. J. Sleep Res..

[39] Hansen A.M., Gullander M., Hogh A., Persson R., Kolstad H.A., Willert M.V., Bonde J.P., Kaerlev L., Rugulies R., Grynderup M.B. (2016). Workplace bullying, sleep problems and leisure-time physical activity: A prospective cohort study. Scand. J. Work. Environ. Health.

[40] Dishman R.K., Oldenburg B., O’Neal H., Shephard R.J. (1998). Worksite physical activity interventions. Am. J. Prev. Med..

[41] Conn V., Hafdahl A., Cooper P., Brown L., Lusk S. (2009). Meta-analysis of workplace physical activity interventions. Am. J. Prev. Med..

[42] Diggle P.J., Heagerty P., Liang K.-Y., Zeger S. (2002). Analysis of Longitudinal Data.

[43] Faraway J.J. (2016). Extending the linear model with R: Generalized linear, mixed effects and nonparametric regression models. Chapman and Hall.

[44] Okano G., Miyake H., Mori M. (2003). Leisure Time Physical Activity as a Determinant of Self-Perceived Health and Fitness in Middle-Aged Male Employees. J. Occup. Health.

[45] Kull M. (2002). The relationships between physical activity, health status and psychological well-being of fertility-aged women. Scand. J. Med. Sci. Sports.

[46] McAuley E., Doerksen S.E., Morris K.S., Motl R.W., Hu L., Wójcicki T.R., White S.M., Rosengren K.R. (2008). Pathways from physical activity to quality of life in older women. Ann. Behav. Med..

[47] Paxton R.J., Motl R.W., Aylward A., Nigg C.R. (2010). Physical activity and quality of life—the complementary influence of self-efficacy for physical activity and mental health difficulties. Int. J. Behav. Med..

[48] Bandura A. (1995). Foreword. Self-efficacy in Changing Societies.

[49] Qiu D., Yu Y., Li R.-Q., Li Y.-L., Xiao S.-Y. (2020). Prevalence of sleep disturbances in Chinese healthcare professionals: A systematic review and meta-analysis. Sleep Med..

[50] Owens J.A. (2007). Sleep Loss and Fatigue in Healthcare Professionals. J Perinat Neonatal Nurs..

[51] Kredlow M.A., Capozzoli M.C., Hearon B.A., Calkins A.W., Otto M.W. (2015). The effects of physical activity on sleep: A meta-analytic review. J. Behav. Med..

